# Does the placebo effect modulate drug bioavailability? Randomized cross-over studies of three drugs

**DOI:** 10.1186/s12952-017-0075-2

**Published:** 2017-05-23

**Authors:** Muhammad M Hammami, Ahmed Yusuf, Faduma S. Shire, Rajaa Hussein, Reem Al-Swayeh

**Affiliations:** 1 Clinical Studies and Empirical Ethics Department, King Faisal Specialist Hospital and Research Center, P O Box # 3354 (MBC 03), Riyadh, 11211 Saudi Arabia; 2 Alfaisal University College of Medicine, Riyadh, Saudi Arabia

**Keywords:** Placebo effect, Bioavailability, Plasma terminal half-life, Pharmacokinetic parameters, Cephalexin, ibuprofen, Paracetamol

## Abstract

**Background:**

Medication effect is the sum of its drug, placebo, and drug*placebo interaction effects. It is conceivable that the interaction effect involves modulating drug bioavailability; it was previously observed that being aware of caffeine ingestion may prolong caffeine plasma half-life. This study was set to evaluate such concept using different drugs.

**Methods:**

Balanced single-dose, two-period, two-group, cross-over design was used to compare the pharmacokinetics of oral cephalexin, ibuprofen, and paracetamol, each described by its name (overt) or as placebo (covert). Volunteers and study coordinators were deceived as to study aim. Drug concentrations were determined blindly by in-house, high performance liquid chromatography assays. Terminal-elimination half-life (t_½_) (primary outcome), maximum concentration (C_max_), C_max_ first time (T_max_), terminal-elimination-rate constant (λ), area-under-the-concentration-time-curve, to last measured concentration (AUC_T_), extrapolated to infinity (AUC_I_), or to T_max_ of overt drug (AUC_Overttmax_), and C_max_/AUC_I_ were calculated blindly using standard non-compartmental method. Covert-vs-overt effect on drug pharmacokinetics was evaluated by analysis-of-variance (ANOVA, primary analysis), 90% confidence interval (CI) using the 80.00–125.00% bioequivalence range, and percentage of individual pharmacokinetic covert/overt ratios that are outside the +25% range.

**Results:**

Fifty, 30, and 50 healthy volunteers (18%, 10%, and 6% females, mean (SD) age 30.8 (6.2), 31.4 (6.6), and 31.2 (5.4) years) participated in 3 studies on cephalexin, ibuprofen, and paracetamol, respectively. Withdrawal rate was 4%, 0%, and 4%, respectively. Eighteen blood samples were obtained over 6, 10, and 14 h in each study period of the three drugs, respectively. ANOVA showed no significant difference in any pharmacokinetic parameter for any of the drugs. The 90% CIs for AUC_T_, AUC_I_, C_max_, AUC_Overttmax_, and C_max_/AUC_I_ were within the bioequivalence range, except for ibuprofen C_max_ (76.66–98.99), ibuprofen C_max_/AUC_I_ (77.19–98.39), and ibuprofen (45.32–91.62) and paracetamol (51.45–98.96) AUC_Overttmax_. Out of the 126 individual covert/overt ratios, 2.0–16.7% were outside the +25% range for AUC_T_, 2.0–4.2% for AUC_I_, 25.0–44.9% for C_max_, 67.3–76.7% for AUC_Overttmax_, and 45.8–71.4% for T_max_.

**Conclusions:**

This study couldn’t confirm that awareness of drug ingestion modulates its bioavailability. However, it demonstrates the trivial effect of blinding in bioequivalence studies and the extent of bio-variability that would be expected when comparing a drug product to itself.

**Trial registration:**

ClinicalTrials.gov identifier: NCT01501747 (registered Dec 26, 2011).

## Background

The placebo effect has been utilized in medical practice since antiquity and continues to be commonly used [1]. Changes over time in the placebo arm of a randomized, double-blind, placebo-controlled trials don’t separate the placebo effect (meaning response) [2] from methodological factors such as regression to the mean, natural course, and the Hawthorne effect. [3] Although it was once argued that if a placebo effect exists it would be of negligible importance [4], under some circumstances, the placebo effect could be clinically important [5, 6] and comparable in size to the drug effect [7–11]. The placebo effect may explain why generic drug products that pass rigorous bioequivalence tests are perceived as less potent (not more potent) than their more expensive branded counterparts [12, 13].

Differences between drug and placebo arms in clinical trials may represent not only drug pharmacological effect but also a drug*placebo interaction effect and thus may underestimate [7, 14] or overestimate [8] pharmacological drug effect. The possibility of a negative interaction effect may explain the clinically trivial effect of antidepressants as deduced from clinical trials [15].

It was suggested since the 1950s that the effect of false belief may countermand the effects of active drugs [16]. A neuro-imaging study showed that alcohol intoxication and expectancy have opposite effects on neuronal activation [17]. The possibility that the drug*placebo interaction effect may involve modulation of drug bioavailability has not been well explored. Theoretically, it is possible that the interaction effect may involve altering gastric emptying, intestinal transit time, or drug elimination. Previously, we conducted a 14 h bioavailability study on 22 volunteers who received caffeine described as caffeine or as placebo in a balanced randomized cross-over design. Mean plasma caffeine levels were consistently lower in the terminal part of the concentration-time curve, caffeine area-under-the-time-concentration curve (AUC) was significantly lower, and plasma caffeine terminal half-life was significantly shorter after receiving caffeine described as placebo [7], suggesting the importance of blinding in bioequivalence studies which compare generic and brand drug products and in clinical trials even with objective endpoints.

The rate and extent of drug bioavailability are commonly evaluated by maximum concentration (C_max_) and AUC to last measured concentration (AUC_T_) or extrapolated to infinity (AUC_I_), respectively, using the non-compartmental method. The rate of bioavailability can be also evaluated by first time of C_max_ (T_max_), the ratio C_max_/AUC_I_, and AUC to T_max_ of reference drug (AUC_Reftmax_). Average bioequivalence (ABE) between a test and reference products of the same drug, the standard worldwide requirement to market generic drug products, is demonstrated if the 90% confidence interval (CI) on their C_max_, AUC_T_, and AUC_I_ geometric mean ratio is in the range 80.00–125.00% [18, 19].

Several concerns have been raised regarding the ABE standards, including, using relatively wide bioequivalence limits and being unable to ensure therapeutic equivalence in all subjects; several individual pharmacokinetic ratios can fall well outside the +20% range despite demonstrating ABE. [18–20] Intra-subject variability is commonly estimated by intra-subject coefficient of variation (CV). Large intra-subject CV can be due to intra-drug variability (first-pass or metabolic variability, gastric emptying, etc.), intra-product variability (tablet to tablet or batch to batch), inter-product variability (generic vs. reference product), or subject-by-product interaction (i.e., the difference between products is not the same across subjects). Large intra-subject variability is especially important for narrow therapeutic index (NTI) drugs, for which individual bioequivalence (IBE) model, 75/75 rule, and intra-subject variability comparisons have been advocated [21–23]. The 75/75 rule requires ≥75% of individual test-reference pharmacokinetic ratios to be within +25%.

We hypothesized that the drug*placebo interaction effect may involve modulation of drug pharmacokinetics. We elected to study two over-the-counter medicines, ibuprofen and paracetamol, because of their expected familiarity to study volunteers (and hence potentially having a placebo effect) and cephalexin as a “negative control” because of its expected unfamiliarity. We were not able to confirm our previous observation in any of the three drugs. However, we used the data to explore the extent of bio-variability that can be observed when comparing a drug product to itself.

## Methods

### Design

Volunteers were consecutively assigned to one of three randomized, two-period, two-sequence, cross-over studies, using cephalexin, ibuprofen, or paracetamol. In each study, the volunteers received one of the three drugs twice, one time described by its name (overt) and one time described as placebo (covert). Wash-out periods and blood sampling frames were drug-specific (Table 1) and extended to ˃7 and ≥5 expected drug plasma half-life, respectively. Expected plasma half-life was about 1 h for cephalexin [24, 25], 2 h for ibuprofen [26], and 2.3 h for paracetamol [27].

**Table 1:** Main characteristics of three randomized, 2-period, 2-sequence, cross-over studies comparing three drugs described by their name or as placebo

Drug name (dose)	Cephalexin (500 mg)	Ibuprofen (400 mg)	Paracetamol (500 mg)
Participants, No. (sex)	41 (M), 9 (F)	27 (M), 3 (F)	47 (M), 3 (F)
Age, mean (SD), year	30.8 (6.2)	31.4 (6.6)	31.2 (5.4)
BMI, mean (SD), kg/m^2^	24.8 (3.1)	25.0 (4.2)	25.2 (3.1)
Adverse events (No.)^a^	Near fainting (1)	None	Localized rash (1)
Washout period, hour	24	24	48
Sampling frame, hour	6	10	14
Assay range, μg/ml	0.50–120	0.25–60	0.10–40

^a^All adverse events were mild and resolved spontaneously

### Participants

We enrolled healthy (based on medical history, complete blood count, renal profile, and liver profile within 30 days), non-pregnant, non-lactating adults (age 18–60 years) with a body mass index (BMI) ≤35 kg/m^2^, who accepted to abstain from taking any medication (including over-the-counter) for 1 week and from smoking, alcohol, and caffeine for 48 h before and throughout each study periods. Subjects with a history of hypersensitivity to the drug to be used or with recent (one week) acute illness were excluded. For menstruating women, the study was conducted 5 to 19 days after the last menstrual period and after obtaining a negative urine pregnancy test.

The study was conducted at the King Faisal Specialist Hospital & Research Center (KFSH&RC), Riyadh from February 2012 through February 2013 after obtaining approval of the KFSH&RC Research Ethics Committee. Volunteers were compensated based on the Wage-Payment model [28] in a prorated manner. The study followed published ethical guidelines on deception use in clinical research [29–32]. A written “consent” (specific to the drug to be administered) was obtained from each volunteer; being told that the study compares the effects of tablets/capsules containing placebo to those containing the particular drug on a new serum marker, that it aims to determine how much of the observed changes in the serum marker is not related to the particular drug, and that they will each receive both the drug and placebo in a random sequence. At the completion of the three studies and after obtaining their monetary compensation, the volunteers were contacted for debriefing on the actual study aim and design and for delayed full consenting.

### Procedures and interventions

The three drugs were purchased from retail pharmacies in Riyadh, Saudi Arabia. Brand name, manufacturer name, batch number, manufacturing date, and expiry date were: Keflex 500 mg, Facta Italy, 000301, 12/2010, and 12/2013 for cephalexin; Ibuprofen 400 mg, Hamol Ltd. UK, 1EE, 5/2010, and 5/2015 for ibuprofen; and Panadol 500 mg, GlaxoSmithKline, 110,216, 2/2011, and 2/2015 for paracetamol.

Few days before the study, the volunteers were reminded to abstain from smoking, alcohol, and caffeine for 48 h, from food for 10 h, and from water for one hour, and to have ≥7 h of good sleep before each study period. Compliance with study instructions was checked before administering the drugs. The drugs were administered (by MMH) with 240 ml of water at room temperature. To enhance the placebo effect, immediately before drug administration, the volunteers were individually briefed and requested to read and sign an additional “consent” document that stated: “As you know, we are doing this study to determine how much of the change in serum marker level that occurs after ingestion of (name and dose of drug) is not related to (name of drug) effect but to placebo effect. This study has two parts. One time you will take (name of drug) and one time you will take a placebo. The placebo is not known to affect the level of the marker. Today you are assigned to take (name of drug or placebo).” Overt drugs were dispensed from the original brand manufacturer bottle, whereas covert drugs were dispensed from a bottle labelled “placebo”. Fasting from food and beverages continued for 4 h post-dosing. However, volunteers were allowed 120 ml water per hour, starting one hour after drug administration. A standardized breakfast and dinner were provided 4 h and about 10 h after drug administration. Meal plans were identical in all parts of the studies. Strenuous physical activity was not permitted during study periods. Volunteers remained ambulatory or seated upright (unless deemed medically necessary) for the first four hours after drug administration and were under continuous observation regarding occurrence of adverse events and compliance with study procedures. In addition, they were directly asked about experiencing any adverse events at the time of last blood collection of each period and at the beginning of period-2.

Eighteen blood samples were obtained before and, at 0.16, 0.33, 0.5, 0.66, 0.83, 1.0, 1.25, 1.5, 1.75, 2.0, 2.5, 3.0, 3.5, 4.0, 4.5, 5.0, and 6 h after cephalexin administration, at 0.25, 0.5, 0.75, 1.0, 1.25, 1.5, 1.75, 2.0, 2.25, 2.50, 3.0, 3.5, 4.0, 5.0, 6.0, 8.0, and 10 h after ibuprofen administration, and at 0.25, 0.5, 0.75, 1.0, 1.25, 1.5, 1.75, 2.0, 2.5, 3.0, 3.5, 4.0, 6.0, 8.0, 10, 12, and 14 h after paracetamol administration. Blood samples were collected in vacutainer tubes, centrifuged (3000 rpm for 10 min) at room temperature within 15 min, and plasma samples were harvested in clean polypropylene tubes and placed immediately at –80 °C.

Drug concentrations were blindly measured by in-house, locally-validated, reversed-phase high performance liquid chromatography (HPLC) assays. [33–35] Limits of quantifications are shown in Table 1. Intra-assay coefficient of variation (CV, standard deviation/mean × 100) and bias (measured concentration/nominal concentration × 100) were ≤3.1% and ≤5.0% for cephalexin, ≤3.8% and ≤7.0% for ibuprofen, and ≤11.6% and ≤14.0% for paracetamol. A typical assay run included a series of 10 standard samples (calibrators), several sets of four quality control samples (concentrations at 1 and 3 times lower quantitation limit and 0.5 and 0.8–0.9 upper quantitation limit), and unknown samples. Standards and quality control samples were distributed throughout the unknown samples. Samples from the two periods of each volunteer were analyzed in the same assay run. Samples with drug concentrations greater than the upper quantitation limit were re-assayed after dilution. Drug concentrations below the lower quantitation limit were assigned zero value. Drug concentrations of missing samples were assigned the value of the average concentration of the two flanking samples in the same period.

### Randomization

Three randomization schedules (one for each drug) were generated (by MMH) using an online program [36]. For each study, volunteers were block-randomized (block size = 2) to one of two sequences (drug described by its name followed by drug described as placebo and vice versa). Assignment was concealed from recruiting study coordinators and potential participants.

### Deception and blinding

Study coordinators and volunteers were deceived regarding study aim and design. Volunteers were deceived in the period when they were given the drug described as placebo. To enhance deception, volunteers were requested not to reveal their assignments to the coordinators. Drug concentrations and pharmacokinetic analysis were performed blinded to assignment.

### Sample size

Calculation of the sample size for each of the three studies was based on the primary analysis of a difference in drug half-life of 10%, type I error of 0.05, type II error of 0.1, and about 10% withdrawal/dropout rate. We estimated that mean and standard deviation (SD) drug half-life are, respectively, 1.0 and 0.21 h for cephalexin [24, 25], 2.0 and 0.30 h for ibuprofen [26], and 2.3 and 0.46 h for paracetamol [27]. The calculated sample size, allowing for withdrawals/dropouts, was 50 for the cephalexin study, 30 for the ibuprofen study, and 50 for the paracetamol study.

### Outcome measures and analysis

The following pharmacokinetic parameters were determined using standard non-compartmental method: AUC_T_ calculated by linear trapezoidal method, terminal rate constant (λ) calculated by linear least-squares regression analysis from a plot of natural log-transformed plasma concentration versus time curve, AUC_I_ calculated as the sum of AUC_T_ plus the ratio of last quantifiable plasma level/λ, AUC_T_/AUC_I_, C_max_ determined directly from the observed data, C_max_/AUC_I_, T_max_ determined directly from the observed data, t_½_ calculated as natural log of 2/ λ,, and AUC to T_max_ of the overt drug (AUC_Overttmax_) calculated by linear trapezoidal method.

The primary outcome measure was t_½_. Secondary outcome measures were the other pharmacokinetic parameters. Outcome measures were evaluated by analysis of variance (ANOVA) after natural log-transformation, except for T_max_. The model included sequence, subjects nested within sequence, period, and intervention (covert vs overt drug administration). Mean square residual error (MSR) was used to test the significance of period and intervention effects. Subjects nested in sequence mean square was used to test the significance of sequence effect. Secondary analysis evaluated covert-overt drug ABE: the 90% CI on the difference between means of log-transformed values was determined (using MSR) and the antilog of the 90% CI limits were compared to the 80.00% and 125.00% bioequivalence limits. The null hypothesis of no placebo effect on drug bioavailability was rejected if the covert-overt drug difference was not significant at the 0.05 level. The null hypothesis of lack of bioequivalence was rejected if the 90% CI was entirely within the 80.00% to 125.00% limits. We also calculated the percentage of individual covert/overt pharmacokinetic ratios that were ˂75% or ˃125% and their mean deviation from 100%.

Pharmacokinetic calculations and statistical analysis were performed (by MMH) on a personal computer using Microsoft Excel (Version 2010) with relevant add-ins (PK Functions for Microsoft Excel, JI Usansky, A Desai, and D Tang-liu, Department of pharmacokinetics and Drug Metabolism, Allergan Irvine, CA, USA) and IBM SPSS Statistics version 21 software (SPSS Inc., Chicago, Ill, USA), respectively. Analyses were not adjusted for multiple comparisons. Two-tailed *p* values are reported.

## Results

**Fig. 1:**
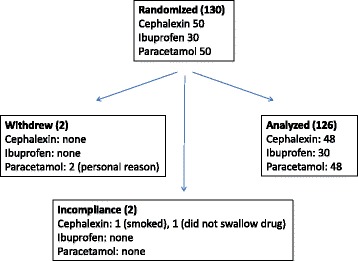
Flow of participants through the study

A total of 130 (Fig. 1) healthy volunteers participated in three two-period, two-sequence, cross-over studies that compared two single oral doses of cephalexin, ibuprofen, or paracetamol, each described by its name (overt) or as placebo (covert). As shown in Table 1, 6–18% of the volunteers per study were females. Mean (SD) age ranged from 30.8 (6.2) to 31.4 (6.6) years and mean BMI from 24.8 (3.1) to 25.2 (3.1) kg/m^2^. As shown in Figure 1, withdrawal rate ranged from 0% (ibuprofen) to 4% (cephalexin and paracetamol). Withdrawal reasons were personal (one volunteer withdrew before period-1 and one after period-2, refusing to provide post-study consent) or incompliance with study procedures (one volunteer smoked during the study and one did not swallow cephalexin tablet). We were able to contact 68 (54%) out of the 127 volunteers who completed the study for post-study debriefing and consenting, all gave full informed consent except one volunteer (noted above), whose data were removed from analysis. Adverse events occurred in 0% (ibuprofen) to 2% (cephalexin and paracetamol) of volunteers (Table 1); all were minor and resolved spontaneously.

**Fig. 2:**
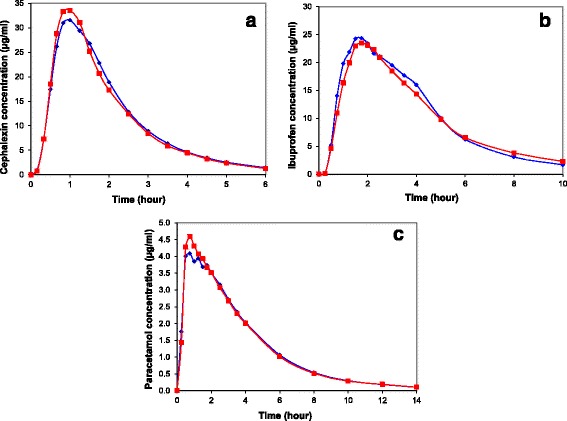
Time-concentration curves of cephalexin (**a**), ibuprofen (**b**), and paracetamol (**c**) described as such (*blue diamonds*) or as placebo (*red squares*). Data represent mean concentrations

**Fig. 3:**
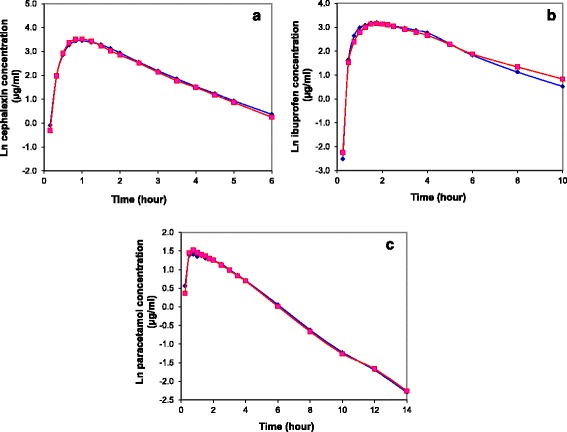
Time-log-concentration curves of cephalexin (**a**), ibuprofen (**b**), and paracetamol (**c**) described as such (*blue diamonds*) or as placebo (*red squares*). Data represent mean natural log-transformed concentrations

Eighteen blood samples were obtained over 6 to 14 h in each study period with a washout interval ranging from 24 to 48 h (Table 1). There were no missing blood samples or interfering plasma peaks in any of the 3 drug assays. Baseline plasma concentrations were below the assay detection limits in all volunteers. In one volunteer in the cephalexin study, cephalexin concentration was not measurable in any sample during one study period (the volunteer, note above, admitted that he did not swallow the cephalexin tablet). The entire data of this volunteer and of the volunteer who did not provide post-study consent were not included in further analysis. Mean untransformed and natural logarithm-transformed concentration-time curve of the three drugs when administered overtly or covertly are presented in Fig. 2 and Fig. 3, respectively. The results are consistent with the results of previous studies on cephalexin [24, 25], ibuprofen [26], and paracetamol [27].

We were able to calculated λ in all analyzed periods. No outlier values for AUC_T_, AUC_I_, or C_max_ were identified/removed from analysis. Main pharmacokinetic parameters of the three drugs when administered overtly or covertly are summarized in Table 2. AUC_T_/AUC_I_ ranged from 93% (ibuprofen) to 97% (cephalexin and paracetamol), indicating adequate sampling frames. MSR from ANOVA analysis and calculated intra-subject CV for each of the 3 drugs are presented in Table 3. The intra-subject CV ranged from 5.5% (paracetamol) to 9.5% (cephalexin) for AUC_I_ and from 23.1% (paracetamol) to 29.8% (ibuprofen) for C_max_. There were no significant (*p*˃0.05) period or sequence effects in any of the three studies.

**Table 2:** Main pharmacokinetic parameters of three drugs described by their name (overt) or as placebo (covert)

	Cephalexin			Ibuprofen			Paracetamol		
Parameter	Overt	Covert	*P* value	Overt	Covert	*P* value	Overt	Covert	*P* value
AUC_T_ (μg.hr./ml)	68.53 ± 26.39	67.20 ± 24.96	0.47	104.38 ± 20.08	101.43 ± 21.82	0.27	18.48 ± 3.98	18.52 ± 3.68	0.62
AUC_I_ (μg.hr./ml)	71.11 ± 28.04	69.61 ± 25.77	0.46	109.40 ± 20.45	109.65 ± 21.54	0.99	19.04 ± 4.11	19.07 ± 3.79	0.65
C_max_ (μg/ml)	40.16 ± 16.51	39.32 ± 17.01	0.68	31.97 ± 6.53	29.17 ± 10.34	0.08	5.72 ± 1.74	6.08 ± 1.62	0.16
T_max_ (hr)	1.11 ± 0.42	1.09 ± 0.49	0.82	1.86 ± 0.90	2.02 ± 1.21	0.58	1.01 ± 0.66	0.94 ± 0.62	0.45
λ (hr ^−1^)	0.68 ± 0.15	0.67 ± 0.13	0.99	0.39 ± 0.06	0.35 ± 0.08	0.052	0.30 ± 0.05	0.30 ± 0.05	0.51
t_½_ (hr)	1.07 ± 0.23	1.07 ± 0.23	0.99	1.85 ± 0.53	2.11 ± 0.74	0.052	2.33 ± 0.35	2.35 ± 0.35	0.51
C_max_/AUC_I_ (hr ^−1^)	0.57 ± 0.12	0.56 ± 0.12	0.86	0.30 ± 0.05	0.27 ± 0.08	0.06	0.30 ± 0.07	0.33 ± 0.10	0.14
AUC_T_/AUC_I_	0.97 ± 0.02	0.97 ± 0.02	0.84	0.95 ± 0.05	0.93 ± 0.08	0.10	0.97 ± 0.01	0.97 ± 0.02	0.74
AUC_Overttmax_ (μg.hr./ml)	17.87 ± 10.91	19.98 ± 15.56	0.73	22.65 ± 9.84	24.69 ± 25.77	0.04	2.51 ± 2.11	3.02 ± 2.90	0.10

Data are unadjusted mean±SD of untransformed values. AUC_T_ is area-under-the-plasma-concentration-time curve from time 0 to last measured concentration. AUC_I_ is area-under-the-plasma-concentration-time curve extrapolated to infinity. C_max_ and T_max_ are first measured maximum plasma level and its time, respectively. λ is terminal elimination constant. t_½_ is plasma half-life. AUC_Overttmax_ is area-under-the-plasma-concentration-time curve to T_max_ under overt drug administration. *P* values were obtained from Analysis of variance (ANOVA) of natural logarithm-transformed values except for T_max._

**Table 3:** Bioequivalence comparison of three drugs described by their name (overt) or as placebo (covert)

Drug name	Cephalexin	Ibuprofen	Paracetamol
AUC_T_			
PE (CI)	98.47% (95.04–102.02)	96.66% (91.79–101.79)	100.62% (98.77–102.50)
MSR	0.011	0.014	0.003
CV	10.5%	11.9%	5.5%
AUC_I_			
PE (CI)	98.54% (95.34–101.83)	99.96% (96.30–103.76)	100.56% (98.71–102.45)
MSR	0.009	0.007	0.003
CV	9.5%	8.4%	5.5%
C_max_			
PE (CI)	97.87% (89.81–106.65)	87.11% (76.66–98.99)	106.79% (98.84–115.37)
MSR	0.063	0.085	0.052
CV	25.5%	29.8%	23.1%
AUC_Overttmax_			
PE (CI)	96.64% (82.17–113.66)	64.44% (45.32–91.62)	71.36% (51.45–98.96)
MSR	0.224	0.642	0.931
CV	50.1%	94.9%	124.0%
C_max_/AUC_I_			
PE (CI)	99.32% (93.00–106.08)	87.15% (77.19–98.39)	106.19% (99.15–113.73)
MSR	0.037	0.076	0.041
CV	19.4%	28.1%	20.5%

AUC_T_ is area-under-the-plasma-concentration-time curve from time 0 to last measured concentration. AUC_I_ is area-under-the-plasma-concentration-time curve extrapolated to infinity. C_max_ is first measured maximum plasma level. AUC_Overttmax_ is area-under-the-plasma-concentration-time curve to T_max_ under overt drug administration. PE is point estimate (antilog of the difference between means of log-transformed data). CI is parametric 90% confidence interval on PE. MSR is mean square residual from analysis of variance. CV is intra-subject coefficient of variation, calculated as 100× (exp(MSR)-1)^0.5^. The number of subjects that were analyzed was 48 for cephalexin, 30 for ibuprofen, and 48 for paracetamol.

### No significant change in the pharmacokinetics of three drugs when described as placebo

*P* values from ANOVA comparing the pharmacokinetic parameters for the three drugs when each was administered overtly or covertly are presented in Table 2. There were no significant differences between the two conditions except for ibuprofen AUC_Overttmax_ (*P* = 0.04). In particular, the *p* value for log transformed λ for the three drugs ranged from 0.052 for ibuprofen to 0.99 for cephalexin.

### Average bioequivalence of three drugs when described by their names or as placebo

**Fig. 4:**
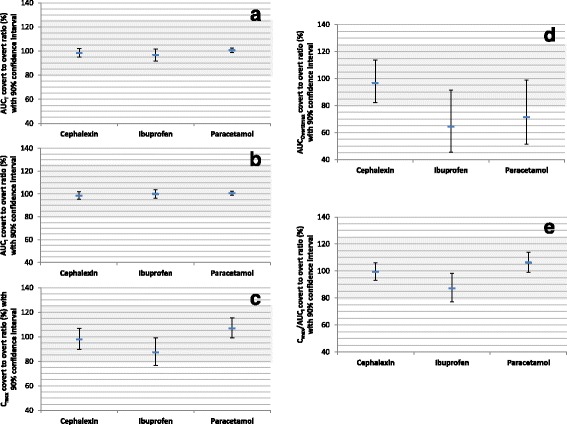
Average bioequivalence evaluation of covert-overt cephalexin, ibuprofen, and paracetamol. Data represent point estimate (antilog of mean covert-overt difference of log-transformed values) and parametric 90% confidence interval. The shaded area indicates the area of bioequivalence (80.00% to 125.00%). **a**, bioequivalence evaluation of area-under-the-concentration-time curve to last quantifiable concentration (AUC_T_). **b**, bioequivalence evaluation of area-under-the-concentration-time curve extrapolated to infinity (AUC_I_). **c**, bioequivalence evaluation of maximum concentration (C_max_). **d**, bioequivalence evaluation of area-under-the-concentration-time curve extrapolated to overt T_max_ (AUC_Overttmax_). **e**, bioequivalence evaluation of C_max_/AUC_I_

Table 3 summarizes the results of bioequivalent analysis comparing AUC_T_, AUC_I_, and C_max_ of the three drugs when they were described by their names compared to when they were described as placebo. There was little difference between the two conditions. Absolute deviation of point estimates from 100% was ≤3.34, ≤1.46, and ≤12.89 percentage points for AUC_T_, AUC_I_, and C_max_, respectively. Further, none of the AUC_T_ or AUC_I_ 90% CIs failed to fall within the 80.00–125.00% bioequivalence limits and only one of the three C_max_ 90% CIs barely failed to do so (76.66–98.99 for ibuprofen). The results are also depicted in Fig. 4. Power analysis revealed that the three studies had a power of ˃0.9 to show average bioequivalence for AUC_T_, AUC_I_, C_max_, and C_max/_AUC_I_, except for ibuprofen C_max_ and C_max /_AUC_I_, were the power was 0.32 and 0.44, respectively.

Table 3 and Fig. 4 also present point estimates and 90% CIs of AUC_Overtmax_ and C_max_/AUC_I_ of the three drugs under the overt and covert conditions. Only ibuprofen AUC_Overtmax_ and C_max_/AUC_I_ and paracetamol AUC_Overttmax_ 90% CIs failed to show bioequivalence and none showed bioinequivalence.

### Individual bioequivalence of three drugs when described by their names or as placebo

**Fig. 5:**
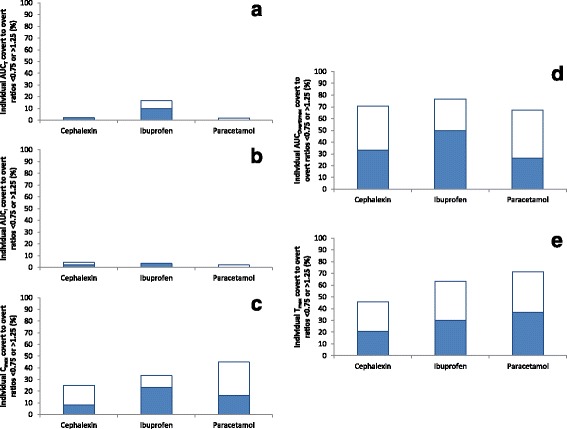
Individual bioequivalence evaluation of covert-overt cephalexin, ibuprofen, and paracetamol. Data represent percentage of individual ratios that are ˂0.75 (closed bars) or ˃1.25 (open bars). **a**, bioequivalence evaluation of area-under-the-concentration-time curve to last quantifiable concentration (AUC_T_). **b**, bioequivalence evaluation of area-under-the-concentration-time curve extrapolated to infinity (AUC_I_). **c**, bioequivalence evaluation of maximum concentration (C_max_). **d**, bioequivalence evaluation of area-under-the-concentration-time curve extrapolated to overt T_max_ (AUC_Overttmax_). **e**, bioequivalence evaluation of time to maximum concentration (T_max_)

The percentages of individual covert/overt AUC_T,_ AUC_I_, C_max,_ AUC_Overttmax,_ and T_max_ ratios that are less than 0.75 or more than 1.25 are presented in Fig. 5.

About 6.9% (least square mean) of the 126 individual ratios were outside the range of 0.75 to 0.125 for AUC_T_ with a range of 2.0% (paracetamol) to 16.7% (ibuprofen), 3.2% for AUC_I_ with a range of 2.0% (paracetamol) to 4.2% (cephalexin), 34.4% for C_max_ with a range from 25.0% (cephalexin) to 44.9% (paracetamol), 71.6% for AUC_Overttmax_ with a range of 67.3% (paracetamol) to 76.7% (ibuprofen), and 60.2% for T_max_ with a range of 45.8% (cephalexin) to 71.4% (paracetamol).

## Discussion

The aim of the study was to examine the hypothesis that the drug*placebo interaction effect involves modulation of drug pharmacokinetics, namely, the knowledge that one is ingesting a drug would alter the pharmacokinetic parameters of the drug. We measured the effect of describing a drug as placebo (covert drug) on the pharmacokinetics of cephalexin, ibuprofen, and paracetamol. The three drugs were selected in part because of their known safety profiles. Ibuprofen and paracetamol were studied because of their expected familiarity (and hence ability to elicit a placebo effect) and cephalexin was used as “negative control” because of its expected unfamiliarity to study volunteers. We designed a two-period, two-sequence, cross-over study for each of the three drugs, with a 90% power to detect a 10% difference in t_1/2_ between overtly and covertly administered drugs. Drug concentrations were blindly determined using in-house HPLC assays and AUC_T_, AUC_I_, C_max_, T_max_, λ, t1/2, and AUC_Overttmax_ were blindly estimated by the standard non-compartmental method. We compared the pharmacokinetic parameters under the two administration conditions using ANOVA, computed 90% CIs on the difference (covert-overt) between the means of their log-transformed values and compared them to the standard bioequivalence range of 80.00% to 125.00%, and calculated the percentages of the untransformed individual pharmacokinetic covert/overt ratios that are outside the +25% range. We found that: 1) there is no placebo effect on any of the pharmacokinetic parameters studied, 2) the two conditions of administering the drugs resulted in bioequivalent profiles, and 3) about 34.4%, 71.6%, and 60.2% of individual covert/overt ratios for C_max_, AUC_Overttmax_, and T_max_, respectively, were outside the +25% range.

### Describing drugs as placebos doesn’t significantly alter their pharmacokinetic parameters

We found no significant differences between the covert and overt conditions in any of the studied pharmacokinetic parameters of the three drugs. This is in contrast to the results of a previous, similarly designed cross-over study on 300 mg caffeine [7], which found that mean plasma caffeine levels were consistently lower in the terminal part of the 14-h concentration-time curve, mean caffeine AUC was significantly lower, and mean plasma caffeine terminal half-life was significantly shorter when caffeine was given covertly. The reason for the discrepancy is not clear. It is possible that the results of the caffeine study were due to chance, especially because the study was exploratory, had a low power (22 subjects), and tested a novel mechanism, which are expected to increase the probability of false discovery, despite having statistically significant results [37, 38]. However, it is also possible that a placebo effect for ibuprofen and paracetamol was not successfully elicited in the current study; other outcomes (for example pain reduction) unfortunately were not examined. There are several modulators of the placebo effect, including conditioning [39], expectancy, suggestion, personality, desire for symptom change, and affective state [40].

### Average bioequivalence of the three drugs under covert and overt conditions

Since no placebo effect was observed on the pharmacokinetic parameters of the three drugs, the volunteers could be considered to have received the same drug product. We thus used the data to explore the extent of bio-variability that can be observed when comparing a drug product to itself. We found that the two conditions of administering the drugs resulted in bioequivalent profiles; only one of the three C_max_ 90% CIs barely failed to show bioequivalence using the strict 80%–125% bioequivalence limits. The outcome of a cross-over bioequivalence study is affected by its sample size and intra-subject variability. In retrospect, power analysis revealed that the ibuprofen study has only a 0.32 power to show bioequivalence for C_max_.

Intra-subject variability can be due to the drug substance itself (being readily affected by physiological variability of the volunteer), product quality variability, analytical variability, or unexplained random variation [41]. In a typical bioequivalence study comparing test and reference drug products, intra-subject variability includes, in addition, variability due to differences between the two products [41]. When a generic drug product is declared therapeutically equivalent to a reference product, it is expected that any difference between the two products should be no greater than the difference between two batches of the reference product. In fact, reviewing the bioequivalence studies of its approved generic products, the US Food and Drug Administration (FDA) found a mean generic-reference deviation of 3.47% for AUC and 4.29% for C_max_ in one study [42] and 3.56% for AUC and 4.35% for C_max_ in another [43]. Commonly, there are several marketed drug products that are linked by a chain of reference [44]; concerns have been raised that reference-bioequivalent generic products may not be bioequivalent to each other. However, secondary analysis of 120 bioequivalence studies on three immunosuppressants and six selected drugs showed a mean generic-generic deviation of 4.5% for AUC_T_ and 5.1% for C_max_ [45]. Interestingly, in the current study, we found that covert-overt deviation was ≤3.34, ≤1.46, and ≤12.89 percentage points for AUC_T_, AUC_I_, and C_max_, respectively; suggesting that most of the deviation observed in typical bioequivalence studies is not related to using two different products.

Bioequivalence studies’ guidelines of regulatory agencies (except for Health Canada (HC)) are silent regarding blinding of study volunteers [18–20]. Our results suggest that failure to blind would not be expected to have a negative impact; even describing the drugs as placebo did not affect their pharmacokinetic parameters.

There is disagreement among the different regulatory guidelines on the bioequivalence criteria for C_max_, whereas some require that the 90% CI should fall within the 80–125% or the 70–143% limits, others require only the point estimate to fall within the 80–125% limits [18–20]. Our results suggest that requiring the 90% CI to fall within the 80–125% limits may be too strict as ibuprofen reference product failed this criteria when compared to itself, despite having a relatively large sample size.

For drugs where time of onset of effect is important, the US FDA and HC recommend that the 90% CI of AUC truncated at reference product median T_max_ or AUC_Reftmax_, respectively, should be within the 80–125% limits [18–20]. Our results indicate that such criteria would be difficult to achieve; only one of the three 90% CIs of AUC_Overttmax_ was within the 80–125% limits.

### Individual bioequivalence of the three drugs under covert and overt conditions

It has been argued that because ABE testing focuses on differences between mean values and relatively neglects differences between variances and subject-by-product interaction, it is possible that, despite establishing ABE, a patient switched from a reference product to a generic product (or vice versa) could be over-dosed or under-dosed and that some patients may have the highest drug exposure values with the reference product and lowest exposure values with the generic product and vice versa [46]. In fact, a bioequivalent study comparing generic and reference cyclosporine products found that 38% of individual C_max_ ratios and 18% of individual AUC ratios were less than 0.80 despite having 90% CI within the 80–125% limits [47].

Our findings that about 6.9%, 3.2%, and 34.4% of individual covert/overt ratios for AUC_T_, AUC_I_, and C_max_, respectively, were outside the +25% range indicate that most of the variation in individual ratios are not related to using different drug products but rather to the volunteers, drug moiety, study setting, drug assay, or random variations. Our findings are consistent with the results of previous studies [48, 49]. A simulation study (assuming inter-subject variability of 20% and intra-subject variability of 10%) found that 11.1% of the reference/reference AUC ratios fell outside the 0.80–1.25 range [48]. Further, in a fully-replicated bioequivalence study on the antiepileptic drug, lamotrigine, 3% and 18% of the generic/generic ratios and 3% and 9% of the reference/reference ratios for AUC and C_max_, respectively, were outside the 0.75–1.25 range [49].

Finally, it could be argued that some patients’ distrust of reference-bioequivalent generic products [12, 13] could be related to generic products’ different onset of action, which in turn may be related to different inactive ingredients or manufacturing processes. However, this is not likely. Onset of a drug effect is for the most part related to its pharmacodynamic characteristics and as shown in the current study, large variations in T_max_ and AUC_Overttmax_ (60.2% and 71.6% of individual ratios, respectively, were outside the ±25% range) can be observed when comparing a product to itself.

### Limitations

The interpretation of the results of this study is limited by the following. 1) Intervention’s administration by an undeceived investigator may have reduced the placebo effect. 2) It is possible that the study setting and the drugs used were not conducive to elicit adequate placebo effect; thus the finding of no difference in pharmacokinetic parameters between the overt and covert conditions may not be related to the placebo effect not modulating drug bioavailability but rather to failure to induce a placebo effect. 3) The protocol-defined aim of the study was to examine if the drug*placebo effect involves modulating drug bioavailability; thus the findings regarding bioequivalence of drug products with themselves are based on post hoc analysis. 4) Our study was not designed to partition intra-subject variability into its various components. Thus, it is not clear how much of the observed variability is due to the drug products themselves (i.e. to drug product quality variability rather than to the drug moiety, random error, etc.).

## Conclusions

This study couldn’t confirm that awareness of drug ingestion can modulate its bioavailability. Although this may be due to inability to elicit adequate placebo effect, the results cast doubt on the concept that the drug*placebo interaction effect may involve modulating drug pharmacokinetics through mechanisms such as altering gastric emptying, intestinal transit time, or drug elimination. On the other hand, the study demonstrates that most of the generic-reference deviation observed in typical bioequivalence studies may not be related to using different products but is inherent in study design and setting, that failure to blind subjects in bioequivalence studies may not negatively impact validity of the results, that the 80–125% bioequivalence limits for C_max_ and AUC_Overttmax_ 90% CI may be too strict, and that considerable intra-subject variability in C_max_, T_max_, and AUC_Reftmax_ would be expected even when comparing a drug product to itself.

## Abbreviations

ABE: Average bioequivalence
ANOVA: Analysis of variance
AUC_T_: Area-under-the-concentration-time-curve to last measured concentration
AUC_I_: Area-under-the-concentration-time-curve extrapolated to infinity
AUC_Overttmax_: Area-under-the-concentration-time-curve to time of maximum concentration (T_max_) of drug given overtly
AUC_Reftmax_: Area-under-the-concentration-time-curve to time of maximum concentration (T_max_) of reference product
BMI: Body mass index
CI: Confidence interval
C_max_: Maximum concentration
CV: Coefficient of variation
FDA: Food and Drug Administration
HC: Health Canada
HPLC: High performance liquid chromatography
IBE: Individual bioequivalence
KFSH&RC: King Faisal Specialist Hospital and Research Center
MSR: Mean square residual error from ANOVA
SD: Standard deviation
T_max_: Time of maximum concentration
Λ: Terminal elimination rate constant
